# Living at depth: ecophysiological condition of *Boreomysis arctica* in autumn and winter in the St. Lawrence estuary and gulf

**DOI:** 10.1093/plankt/fbae022

**Published:** 2024-05-16

**Authors:** Gesche Winkler, Jory Cabrol, Réjean Tremblay

**Affiliations:** Institut de Sciences de la Mer, Université du Québec à Rimouski, Québec-Océan, 310 Allée des Ursulines, G5L3A1, Rimouski, Quebec, Canada; Maurice Lamontagne Institute, Fisheries and Oceans Canada, 850 Rte de la Mer, G5H 3Z4, Mont-Joli, QC, Canada; Institut de Sciences de la Mer, Université du Québec à Rimouski, Québec-Océan, 310 Allée des Ursulines, G5L3A1, Rimouski, Quebec, Canada

**Keywords:** ecophysiological condition, lipids, trophic ecology, Boreomysis arctica, St. Lawrence

## Abstract

Mysids, besides krill, play a significant role in energy transfer and carbon sequestration. The ecology of coastal species is better understood than that of deep dwelling species such as *Boreomysis arctica*. The objectives of this study were to quantify spatiotemporal variations in body condition and the trophic level of *B. arctica* in autumn and winter, under sea-ice conditions in the St. Lawrence system, using a multimarker approach. We sampled along a 1000 km transect. Mean abundances in winter were higher in the estuary compared to the Gulf of St. Lawrence. Body condition, measured as total lipid content, was higher in winter than in autumn. Lipids of *B. arctica* were mainly composed of wax esters, thereby *B. arctica* is richer in energetic lipids compared to the three dominant krill species. We also observed seasonal differences in the trophic level of *B. arctica,* revealing carnivorous behavior in autumn compared to omnivory in winter*.* High intra-specific variability in both energetic strategy and feeding behavior was found that is potentially due to opportunistic feeding. Energy rich reserves suggest that *B. arctica* could act as a valuable prey for both benthic and pelagic consumers and thus playing a key role in bentho-pelagic energy transfer.

## INTRODUCTION

**Fig. 1:**
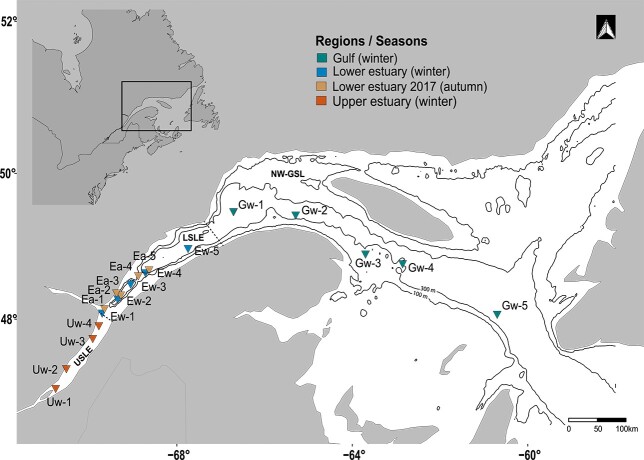
Map of the estuary and the Gulf of St. Lawrence, including sampling stations in autumn 2017 and winter 2018. The map was drawn with R v4.1.1, using open source data. Bathymetry was provided by the National Oceanic and Atmospheric Administration (NOAA) throughout the marmap package.

Mysids are a major component of estuarine and coastal zooplankton communities due to the high abundance, biomass and widespread distribution. They occupy an important link in marine food webs by holding a key position between benthos, plankton and nekton (Tattersall and Tattersall, 1951; Mauchline, 1980; Fulton, 1982). About 780 species within 120 genera of Mysidacea are known (Mauchline, 1980). Most of the species are marine and predominantly shallow water organisms, living in close vicinity to the sediment surface. The ecology of neritic mysids is relatively well documented, whereas ecological information for deep-dwelling species is very scarce. One of these species, *B. arctica*, has a wide distribution throughout the Northern Hemisphere (Mauchline, 1980; Brunel *et al.*, 1998), mostly in the North Atlantic and the Mediterranean Sea (Macquart-Moulin, 1993; Cartes and Sorbe, 1998), occupying meso- and bathypelagic habitats down to 1400 m. In the Estuary and the Gulf St. Lawrence (EGSL), *B. arctica* is found in areas of depths between 200 and 500 m. It was occasionally observed to explore the water column at 150 m, but was never found in surface waters (0–50 m; Harvey *et al.*, 2009). In the Lower St. Lawrence Estuary (LSLE) and the north-western GSL (Fig. 1), *B. arctica* was consistently found in samples from the deep channel habitats with high mean abundances up to 125 ind.m^−2^ (Descroix *et al.*, 2005). These abundances suggest that *B. arctica* is an important contributor to the macrozooplankton community besides the three dominant krill species, *Meganycthiphanes novegica*, *Thysanoessa raschii* and *T. inermis* also occurring in very high abundance in the St. Lawrence ecosystem. While the distribution and population dynamics (e.g. Simard *et al.*, 1986; Simard and Lavoie, 1999; Maps *et al.*, 2014, 2015; Plourde *et al.*, 2016; Benkort *et al.*, 2019) and more recently the ecology (Plourde *et al.*, 2011; Ollier *et al.*, 2018; Cabrol *et al.*, 2019a, b, 2020; Cope *et al.*, 2021) of the three krill species are well documented, the ecology of *B. arctica,* especially in the EGSL, is mostly unknown. In contrast to the three krill species, no diel vertical migration pattern to food rich surface layers has been observed (Harvey *et al.*, 2009). As a consequence, *B. arctica* in the St. Lawrence system will experience relatively constant physicochemical conditions during its entire life cycle (Galbraith *et al.*, 2020) without direct access to freshly produced phytoplankton biomass in the photic zone. Therefore, *B. arctica* seems to be dependent on the organic matter flow from the surface layer into the deep water and/or on the zooplankton residing in these deep zones to acquire food. Even, if *B. arctica* lives <150 m under relatively stable temperature and salinity conditions year-round, the upper part of the water column exhibits high seasonal contrasts, including ice-cover, negative water temperatures and low light regime in winter reducing phytoplankton productivity compared with summer temperatures and good light conditions throughout spring to autumn (Galbraith *et al.*, 2020; Blais *et al.*, 2021). Furthermore, local upwelling in the LSLE induces a prolonged phytoplankton production, resulting in chlorophyll *a* concentration of > 1 mg.m^−3^ from May to October (Plourde *et al.*, 2011; Blais *et al.*, 2021). In contrast, the phytoplankton productivity in the Gulf of St. Lawrence is more patchy in space and time (Blais *et al.*, 2021), strongly affecting local carbon export to the bottom and consequently food availability and quality to deep-dwelling species.

In the Mediterranean Sea, where most of the knowledge on the feeding ecology of *B. arctica* has been documented, *B. arctica* is a generalist and opportunistic feeder, due to its wide range of food sources (Cartes, 2011). This species has been described as a non-selective omnivorous-carnivorous feeder (e.g. Cartes and Sorbe, 1998; Cartes, 2011). Based on gut contents and fatty acid trophic markers *B. arctica* seems to feed on phytodetritus up to large zooplankton species, including dinoflagellates, tintinnids, cnidarians and mesozooplankton such as *Calanus* species. Despite this large range of prey size, crustaceans seemed to be the main prey, suggesting a more carnivorous than detritus based feeding behavior (Mauchline, 1980; Cartes and Sorbe, 1998; Cartes, 2011). Like many macrozooplankton species exposed to variable food supply, *B. arctica* accumulate energetic reserves in the form of lipids. These are stored as neutral lipids, mostly build as triacylglycerol and/or wax esters that can be quickly metabolized for short-term energy requirements (Lee *et al.*, 2006). According to Cartes (2011) the lipid content of *B. arctica* is higher during winter than summer. The same dynamics were also observed for *Thysanoessa* species in the St. Lawrence. For krill, such dynamic are expected to be the result of an energetic strategy to partially limit the need for food when primary production is at its lowest, potentially limiting intra- and interspecific competition when resource availability is low (Cabrol *et al.*, 2019a, 2019b). However unlike krill species little is known about the feeding habits and the physiological strategies of *B. arctica* and how this species might acclimate to local variation of food supply, especially in winter in the St. Lawrence system.

This study aims to quantify spatiotemporal variations of trophic ecology of *B. arctica* focusing on the total lipid content and composition as well as their trophic level along a transect of 1000 km from the upper part of the St. Lawrence estuary to the Cabot Strait in the Gulf of St. Lawrence with a special focus on the winter season. The specific objectives were to examine the spatial and seasonal variation of (i) energetic reserves (e.g. lipid dynamics) of *B. arctica,* and (ii) its trophic position to explore potential changes. We used a multimarker approach, combining lipid class analyses (Parrish and Ackman, 1985) and stable isotope analyses (Fry, 2006). In the present study, the physiological conditions of individuals of *B. arctica* were evaluated by measuring the total lipid content and lipid composition using thin layer chromatography, while trophic levels were calculated from nitrogen stable isotope ratios. These results were further compared with the three dominant krill species from the same area (Cabrol *et al.*, 2019a, 2019b). In addition to serving as a baseline, these results will help to better understand the role of this deep-dwelling mysid species in the bentho-pelagic coupling in the subarctic St. Lawrence ecosystem.

## METHODS

### Sampling

*B. arctica* was collected in autumn 2017 and winter 2018 on board the research vessel R.V. Coriolis II and the Canadian coast guard icebreaker CCGS Amundsen, respectively. In autumn 2017 the study covered four sites in the LSLE as part of two distinct expeditions (Fig. 1). In winter 2018, sampling was extended into the GSL, covering a transect of 14 stations (>1000 km) from Québec to Cabot Strait (Fig. 1). *B. arctica* was collected with a 1 m diameter ring net with 202 μm mesh size equipped with a strobe light (Jacknet), however, no open-closing device was attached to the net. In autumn 2017, oblique tows were taken by lowering the net in an angle of 60° and a speed of 1 m.s^−1^ down to 10 m off bottom and retrieving it in the same manner. Due to malfunctioning of the flowmeter, only a qualitative analysis of the zooplankton has been performed. In winter 2018, vertical hauls were carried out from 5 m off bottom to the surface at a speed of 0.5 m.s^−1^. *B. arctica* were counted and sorted on board. For every station specimens were individually frozen at −80°C for lipid class and stable isotope analyses, respectively (see Table 1 for sample size). Water samples for particulate organic matter (POM) were collected at two discrete depths (10 m and 10 m from the bottom) using a rosette sampler system equipped with 12 L Niskin-type bottles. However, only the bottom sample was used in the present study. To determine stable isotopes of POM, two technical replicates of 1 to 2 L of water were filtered through pre-combusted and pre-weighed 21 mm GF/F filters. The filters were stored at −80°C before analyses.

**Table 1:** *Station coordinates of sampling stations in the Estuary and Gulf of St. Lawrence in autumn 2017 and winter 2018, number of* Boreomysis arctica *captured and/or analyzed for lipid classes and stable isotopes*

Mission	Year	Season	Station	Latitude	Longitude	Station depth (m)	Total caught (n)	Lipid class analyses—(n)	Stable isotope analyses (n)
Coriolis II	2017	Fall	Ea-1	48° 10.550	69° 29.750	226	no count	5	6
			Ea-2	48° 23.300	69° 15.100	309	no count	6	21
			Ea-3	48°22.399	69° 09.712	260	no count	4	10
			Ea-4	48°37.729	68° 45.184	338	no count	0	10
			Ea-5	48°42.937	68° 31.332	345	no count	6	0
CCGS	2018	Winter	Uw-1	47°01.937	70°45.914	18	0	0	0
Amundsen			Uw-2	47°18.776	70°31.005	22	0	0	0
			Uw-3	47°44.145	69°54.871	143	0	0	0
			Uw-4	47°55.012	69°46.741	127	0	0	0
			Ew-1	48°06.472	69°34.143	125	0	0	0
			Ew-2	48°18.305	69°12.839	196	15	6	10
			Ew-3	48°30.703	68°55.196	272	47	3	10
			Ew-4	48°40.0950	68°34.9286	330	66	5	10
			Ew-5	49°00.111	67°36.643	295	23	5	9
			Gw-1	49°31.983	66°10.723	340	16	6	10
			Gw-2	49°27.569	65°09.429	370	9	3	4
			Gw-3	48°55.349	63°34.081	330	16	5	10
			Gw-4	48°47.316	62°43.452	340	3	0	0
			Gw-5	48°05.515	60°34.129	420	1	0	0
Total *B. arctica* analyzed								54	110

### Lipid class analyses

Lipid analyses were performed on the entire individual. Frozen individuals of similar size range (mean total length of 1.92 ± 0.35 cm) were directly extracted in dichloromethane:methanol by grinding using a modified Folch procedure (Chen *et al.*, 1981) as described in detail in Parrish *et al.* (1999). Then, lipid content and class composition were determined using flame ionization detection system using silica gel-coated chromarods (S-V Chromarods; Shell-USA; see Parrish, 1987 for details). Every lipid extracts were scanned using an Iatroscan (Mark-VI, Iatron Laboratories) to separate aliphatic wax esters, ketones, triacylglycerols, alcohols, sterols, acetone mobile polar lipids, and phospholipids (Parrish *et al.*, 1999). However, the method used cannot differentiate steryl esters from wax esters (Parrish, 2013) and are therefore grouped in the term SE-WE and we referred in the following to this group as wax esters for simplification. In fact, in marine animals, a many store lipids as wax esters (Budge *et al.*, 2006) justifying this simplification. For each lipid class, Sigma standards were used for post quantification. Chromatograms were analyzed using the integration software Peak Simple version 3.2 (SRI). Total lipids are expressed as μg.mg^−1^ of wet weight and correspond to the sum of all classes (Parrish, 2013). Lipid classes were expressed as relative concentration in percent of the total lipids (% of total lipids).

### Stable isotope analysis

To estimate trophic levels, stable isotopes of nitrogen (δ^15^N) of *B. arctica* and POM were determined by a continuous-flow Isotope Ratio Mass Spectrometry using a Deltaplus XP mass spectrometer (ThermoScientific) coupled with an elemental analyzer COSTECH 4010 (Costech Analytical). Certified reference material Sorghum Flour Standard OAS (B2159) and High Organic Sediment Standard OAS (B2151) and two in-house standards Caffeine and Mueller Hint Broth were used as internal standards for nitrogen calibration. Data analysis was performed with Isodat 3.0. Analytical error of measurement was 0.2‰ for δ15N of *B. arctica* and *Calanus* spp. and was 0.4‰ for POM-filters. Stable isotopes are expressed in δ notation as the deviation from international standards in parts per thousand according to the following equation:


(1) \begin{equation*} \mathrm{\delta} \mathrm{X}=\left[\left({\mathrm{R}}_{\mathrm{sample}}/{\mathrm{R}}_{\mathrm{standard}}\right)-1\right]\ast 1000 \end{equation*}

where X is ^15^N, and R is the corresponding ratio ^15^N/^14^N.

#### Trophic level determination

The trophic level (TL) was calculated using the equation of Post (2002):


(2) \begin{equation*} \mathrm{TL}=\left[\left({\mathrm{\delta}}^{15}{\mathrm{N}}_{\mathrm{consumer}}\hbox{--} {\mathrm{\delta}}^{15}{\mathrm{N}}_{\mathrm{base}}\right)/\Delta \mathrm{n}\ \right]+\mathrm{\lambda} \end{equation*}

Where δ^15^N_consumer_ = δ^15^N of *B. arctica*, δ^15^N_base_ = δ^15^N of POM near to the bottom and λ is the TL of the baseline used, i.e. TL = 1 for mean POM per season and region. Δn is the estimated trophic discrimination factor for nitrogen. We used deep-water POM signatures that were averaged for each region and season (please refer to Fig. 5 for isotopic values) because *B. arctica* is known to be a deep dwelling species. As no trophic discrimination factors were available for *B. arctica* a trophic discrimination factor of 2‰ was chosen for nitrogen according to McCutchan *et al.* (2003) and Chew *et al.* (2012).

### Statistical analyses

To test differences in lipid contents and trophic levels within and between the two seasons and among sampling sites, univariate two-way PERMANOVAs were performed with “season” (2 levels) and “station” (11 levels) as factors, using an Euclidean distance, type III error structures and 9 999 permutations (Anderson *et al.*, 2008). To determine if the lipid class composition varied between seasons and sampling regions, a multivariate two-way PERMANOVA was carried out, based on an Euclidian distance matrix with untransformed data (Clarke *et al.*, 2014; Clarke and Gorley, 2015). In addition, pairwise multiple comparison tests were used to identify differences among factors when necessary. A non-metric dimensional scaling (n-MDS) was used on the Euclidian distance matrix to visualize inter-individual, inter-station and seasonal variation of lipid class composition. The similarity percentages (SIMPER) procedure was performed on untransformed data to identify specific lipid classes explaining the most important dissimilarities of significant pairwise comparisons. All the tests mentioned above were performed on PRIMER version 7.0.21 (Clarke and Gorley, 2015) with PERMANOVA (Anderson *et al.*, 2008).

## RESULTS

### Abundance and distribution of *B. arctica*

**Fig. 2:**
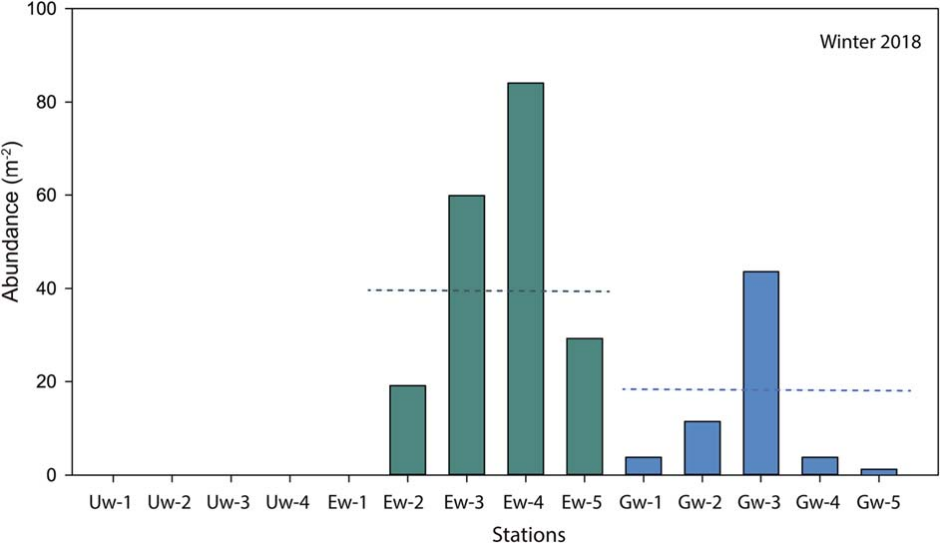
Abundance of *Boreomysis arctica* sampled during winter 2018 in the lower estuary (Ew) and the Gulf (Gw) of St. Lawrence. Data for 2017 were not included due to malfunctioning of the flowmeter. Dashed line represents the mean of each region.

In winter, *B. arctica* were absent in the upper St. Lawrence Estuary (USLE) and at the head of the Laurentian Channel (Ew-1), but were found downstream throughout the lower St. Lawrence Estuary (LSLE) and the Gulf of St. Lawrence (GSL; Fig. 2). Mean abundances were two times higher in the LSLE (40 ind. m^−2^) than in the GSL (Fig. 2), however not significantly different (t-test, *P* = 0.06). Highest abundances of 60 and 84 ind.m^−2^ were found at Ew-3 and Ew-4, respectively, in the LSLE, whereas most of the stations in the GSL showed abundance, <15 ind.m^−2^ (Fig. 2).

### Total lipid content and lipid class composition

**Fig. 3:**
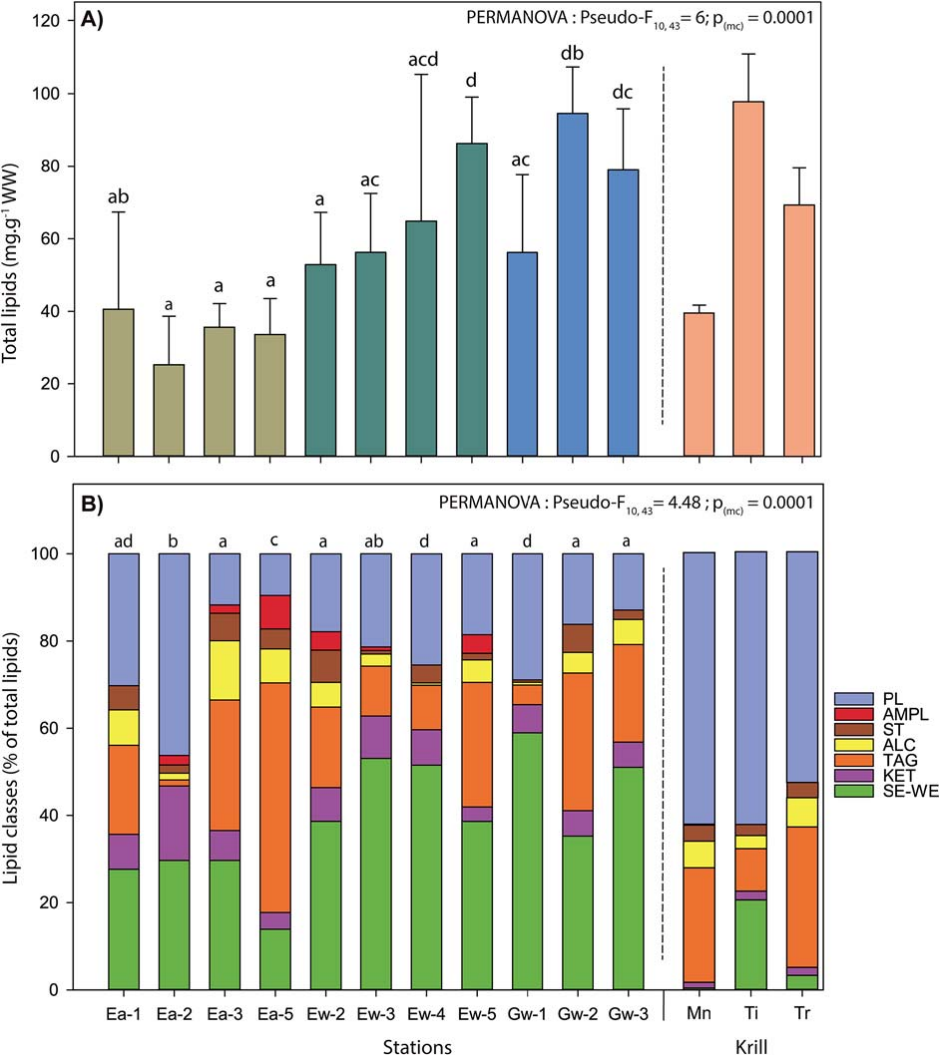
Lipid content in mg.g^−1^ of WW (**A**) and lipid classes in percent of total lipids (**B**) for *Boreomysis arctica* sampled in the estuary and the Gulf of St. Lawrence in autumn 2017 and winter 2018. Note that lipid content and composition of the dominant krill species have been added for comparison and were extracted from Cabrol *et al.*, 2019a sampled during winter 2015. Mn = *Meganyctiphanes norvegica*; Ti = *Thysanoessa inermis* and Tr = *Thysanoessa raschii.*

Total lipid content of *B. arctica* was higher in winter than in autumn (Fig. 3A). Spatial variability of total lipid content was high, but in winter 2018 lipid content generally increased from the LSLE to the GSL showing the highest mean total lipid content at Gw-2 of 94.55 ± 12.78 mg.g^−1^ WW (Fig. 3A). Compared to the three dominant krill species in winter, highest mean lipid contents of *B. arctica* were most similar to that of *T. inermis* and higher than that of *T. raschii* and *M. norvegica* (Fig. 3A). However, when comparing the mean winter lipid content over all stations of 65.44 ± 20.00 mg.g^−1^ WW of *B. arctica*, it is comparable to that of *T. raschii*.

Lipid composition of *B. arctica* was highly variable among individuals and among stations (Fig. S1). It differed among stations in autumn, while in winter it was more homogenous throughout the EGSL (Fig. 3B). In winter, lipid composition of most of the individuals tend to be comparable with a dominance of wax esters (SE-WE) and similar proportions of triacylglycerol (TAG) and polar lipids (PL) (Fig. 3B). The differences between autumn and winter were mainly related to different proportions of SE-WE, TAG and PL (71–88% cumulative contribution—SIMPER), showing high proportions of TAG and PL in autumn (26% and 25% respectively, LSLE) compared to high contribution of SE-WE in winter (44% LSLE and 51% GSL).

### Trophic level of *B. arctica*

**Fig. 4:**
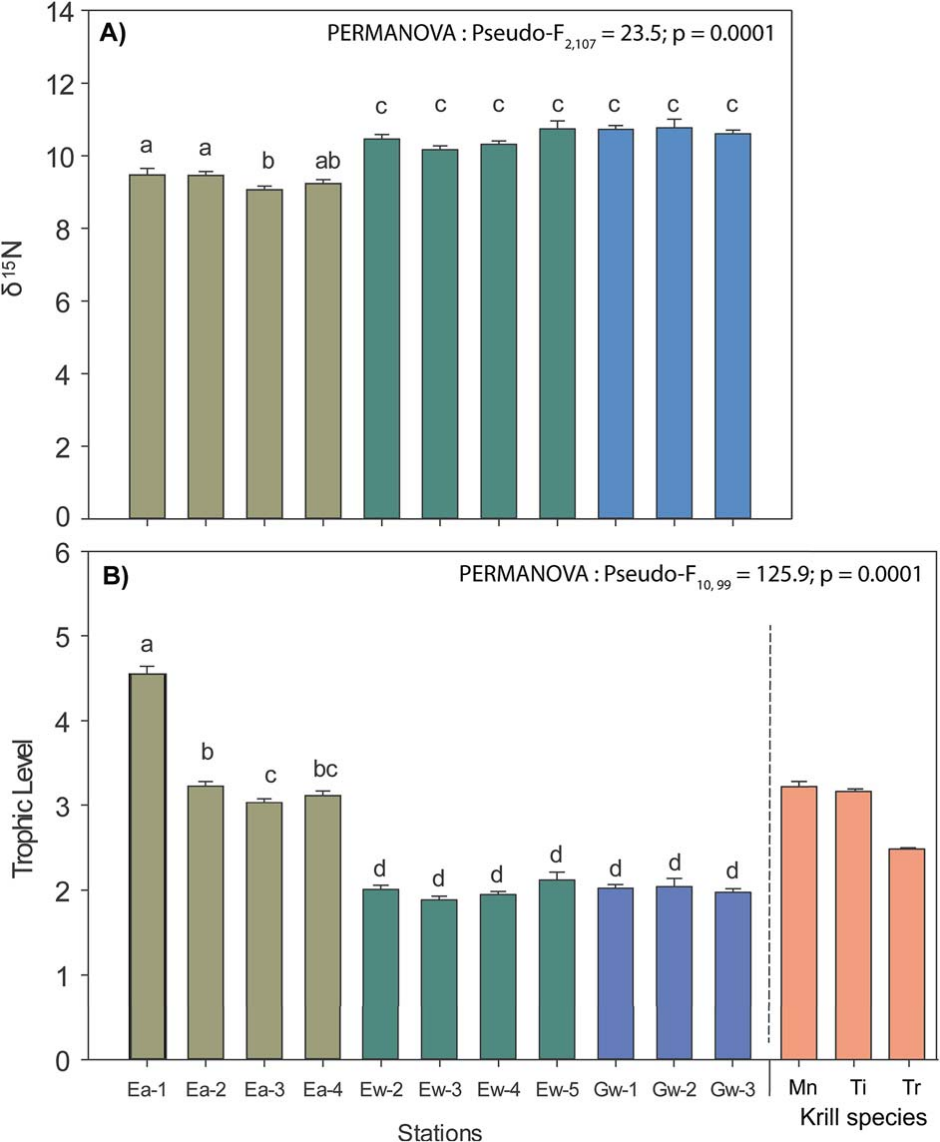
Nitrogen stable isotopes (**A**) and trophic level (**B**) of *Boreomysis arctica* in autumn 2017 and winter 2018 in the estuary and the Gulf of St. Lawrence. Note that trophic levels of krill sampled in the LSLE during winter 2015 were added for comparison (Cabrol *et al.*, 2019a). LSLE = Lower St. Lawrence estuary; Mn = *Meganyctiphanes norvegica*; Ti = *Thysanoessa inermis* and Tr = *Thysanoessa raschii.*

**Fig. 5:**
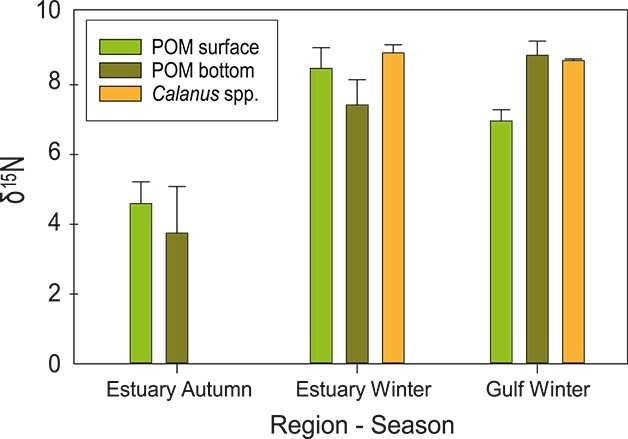
Mean nitrogen stable isotopes (δ^15^N) of particulate organic matter (POM) from the surface and the bottom layer, as well as of *Calanus* spp. in the estuary in autumn 2017 and winter 2018 and the Gulf of St. Lawrence in winter 2018.

The trophic levels of *B. arctica* varied spatially and temporally (Fig. 4A and B). More depleted δ^15^N ratios were found in autumn compared to winter (Fig. 4A), whereas the trophic levels of *B. arctica* showed a contrasting pattern, being higher (3.2 to 4.3) in autumn than in winter (2.0 to 2.7; Fig. 4B). Compared to krill species in the EGSL in winter, *B. arctica* was positioned lower than all three krill species in winter (Fig. 4B). Lower trophic levels (Fig. 5), POM showed enriched δ^15^N ratios of > 2‰ in winter compared to autumn (ANOVA, F_df2_ = 12.455, *P* < 0.001), that were comparable with zooplankton, the calanoid copepod *Calanus* spp. (ANOVA, F_df3_ = 0.714, *P* = 0.556).

## DISCUSSION

### Abundance and distribution of *B. arctica*

As expected, *B. arctica* were absent from shallow and brackish part of the St. Lawrence Estuary. As *B. arctica* has never been observed at depth shallower than 150 m in the LSLE (Harvey *et al.*, 2009), results suggest that *B. arctica* does not pass the shallow sill (40 m) at the head of the Laurentian Channel to migrate into the USLE. In winter, *B. arctica* were found consistently throughout the LSLE and the GSL, showing a tendancy of higher abundance in the LSLE than in the GSL. These abundances suggest that *B. arctica* is an important contributor to the macrozooplankton community besides the three dominant krill species, *Meganycthiphanes novegica, T. raschii* and *T. inermis* also occurring in high abundance in the St. Lawrence ecosystem (Harvey *et al.*, 2009, McQuinn *et al.* 2015). Unfortunately, due to the flowmeter breakdown abundance data in autumn 2017 were not available, preventing any comparison between the two seasons. In 2018, the winter abundances were lower than mean abundances of spring and fall combined with values of 124 ± 10 ind.m^−2^ and 128 ± 7 ind.m^−2^ found in the early 2000s in the LSLE and the north western GSL, respectively (Descroix *et al.*, 2005). Theses authors showed that abundances of *B. arctica* did not vary significantly among years and seasons either in the LSLE or the GSL (Descroix *et al.*, 2005). Data availability is too limited to determine if lower abundances are related to season or potentially to changes in time over the 20 years between the two sampling periods. Over these 20 years, the physico-chemical conditions of the deep-water habitat have, however, changed. Water temperature has increased around 1 to 2°C (Galbraith, 2006; Galbraith *et al.*, 2020) and oxygen condition has severely deteriorated with the increase of the hypoxia level over the last 80 years (Gilbert *et al.*, 2005; Jutras *et al.*, 2020, 2023). This environmental modification is expected to have consequences for the biology and the life cycle of the deep dwelling mysid *B. arctica*, but without proper knowledge on physiological tolerance to temperature and oxygen of this species, this potential impact cannot be estimated.

### Body condition

We estimated physiological body condition in terms of total lipid content. Lipid content of *B. arctica* showed temporal and spatial variability, being higher in winter than in autumn. Bamstedt (1977) found in the Norwegian Korsfjorden that temporal lipid dynamics in *B. arctica* revealed highest lipid percentages of dry weight from October to February and lowest values in early June. Differences of lipid dynamics of *B. arctica* between these two ecosystems may be due to particular climatologies of environmental parameters that may have an influence on the phenology of *B. arctica*. It has been suggested that *B. arctica* in the Mediterranean produce at least two generations per year (Cartes and Sorbe, 1998), showing a peak of recruits in late winter-early spring and one in autumn. Thus, the low lipid content in autumn individuals of the EGSL, might indicate a loss in energy due to reproduction, whereas high lipid content in winter (February) might be indicative of a pre-reproduction period before late winter–spring spawning occurs. However, this explanation should be taken with care, as neither sex nor the developmental stage of the *B. arctica* used in the present study is known.

Compared to the three dominant krill species, lipid dynamics in *B. arctica* seemed to be similar to *Meganycthyphanes norvegica,* showing low lipid content in autumn and highest lipid content in winter (Cabrol *et al.*, 2019a, b). However, the total lipid content in *B. arctica* in winter was higher than that of *M. norvegica,* and similar to levels observed for *Thysanoessa* species. Lipid dynamics are distinct from the three krill species, *B. arctica* accumulating a higher amount of energy from autumn to winter than *M. norvegica.* This stands in contrast to the energetic reserve utilization over winter when compared to both *Thysanoessa* species (Cabrol *et al.*, 2019a). This dynamic also suggests that *B. arctica* is able to acquire enough food from autumn to winter to store energetic lipid reserves. Results of lipids class composition are supporting this hypothesis, as the proportion of energy reserve lipids, composed mainly by wax esters (SE-WE) and triacylglycerol (TAG) in *B. arctica*, were higher in winter than in autumn. The variability in lipid composition of *B. arctica* among stations was higher in autumn when compared to the winter throughout the EGSL. In winter, lipid composition of all the individuals tends to be comparable with a dominance of SE-WE and similar proportions of TAG and polar lipids. The spatial variability in autumn might be related to spatial heterogeneity in food supply and/or food selectivity, resulting in different nutritional histories increasing the variability of the physiological condition among individuals. For example, *B. arctica* at Ea-2 had the lowest lipid content characterized by large TAG depletion and a high proportion of polar lipids, regulating membrane composition (Sargent *et al.*, 1993; Tocher *et al.*, 2008). This depletion of energy reserves indicates weak physiological condition that could be related to energy loss through reproduction, stressful conditions or dying individuals. In contrast, *B. arctica* collected at Ea-5 showed similar low total lipid content, but largely higher proportion of TAG, which might have been acquired by feeding on a high proportion of phytoplankton and/or zooplankton as seen in krill species (Cabrol *et al.* 2019a, b). Cartes (2011) also suggested the presence of a high inter-individual variability in food selection even among individuals collected at the same sampling site, indicating that differential feeding strategies might occur among co-occurring specimens. Accordingly, our results stress the importance of the large intraspecific variability in both energy content and lipid composition between *B. arctica* individuals, particularly at some stations. In these sites, inter-individual variations within *B. arctica* were similar and even higher to the ones found among the three dominant krill species of the northern Atlantic (Cabrol *et al.*, 2019a). High intra-specific variability is a common feature of opportunistic species (Bolnick *et al.*, 2003; Cabrol *et al.*, 2019a, b, 2021). However, such high levels suggest that individuals seem to acclimate to face adverse condition in food supply, as the variability observed probably results from distinct trophic and/or life histories among individuals. As a consequence, physiological condition estimated by lipid content and/or composition and related to their fitness could be expected to vary from one individual to another. In winter, feeding conditions might be more restricted and homogeneous in terms of availability and diversity, reducing the inter-individual physiological condition of *B. arctica*. Early stages and small copepod species abundance might be strongly reduced in winter, whereas diapausing stages of large copepods such as *Calanus* spp. might be easily available in the deep-water layer. Diapausing *Calanus* spp. are non-migrating stages that aggregate in the warmer deep-water layer (150–300 m) compared to the upper freezing surface layer from autumn to spring (Plourde *et al.*, 2002, 2003). This increases the encounter probability of *Calanus* spp. with *B. arctica* during winter. Diapausing *Calanus* spp. have accumulated high amounts of reserve lipids during summer and autumn, mainly in the form of WE (Scott *et al.*, 2002), to fulfill the reduced metabolic demand of the dormancy over winter (Maps *et al.*, 2010, 2011, 2012). Thus, diapausing *Calanus* spp. could be the most important prey, supplying *B. arctica* with WE and might explain the net increase of lipid content as well as the proportion of WE (~1.5 to 2 times) observed during the winter at all stations.

### Trophic level of *B. arctica*

Although *B. arctica* is present throughout the Laurentian Channel of the EGSL, the position in the pelagic food web and the trophic relationships of *B. arctica* are not known. This species is expected to be dependent on resource transport into the deep water of the Laurentian Channel through the sedimentation of POM (phytoplankton, detritus) and diel vertical migrating or diapausing zooplankton. Thus, feeding conditions will be strongly dependent on spatial–temporal variation in primary and secondary production. In the present study, we determined the trophic levels of *B. arctica* in autumn 2017 and winter 2018 throughout the Laurentian Channel by δ^15^N ratios and confirmed spatial and temporal variations with depleted δ^15^N ratios in autumn compared to winter. However, the trophic levels of *B. arctica* showed a contrasting pattern, as the trophic position showed higher levels in autumn, compared with winter, including high spatial variability in the EGSL. These results suggest that *B. arctica* was mostly carnivorous in autumn, whereas it exhibits a more omnivorous feeding behavior in winter. *B. arctica* in the western Mediterranean and the Algerian Basin showed relatively low δ^15^N ratios of 6.5 ± 0.8 and 6.3 ± 0.8, respectively, when compared to the suprabenthic community suggesting an omnivorous filter feeding (Madurell *et al.*, 2008; Fanelli *et al.*, 2009). Evidence of an omnivorous to carnivorous feeding habit was observed based on FAs markers (Cartes, 2011) and gut content analyses (Cartes and Sorbe, 1998) in the Mediterranean Sea. Considering the limited amount of phytoplankton available in winter, carnivory would be the strategy of choice. However, we found the opposite. This might be partly due to the calculation of the trophic level based on δ^15^N of POM. POM showed enriched δ^15^N ratios of > 2‰ in winter compared to autumn, indicative of highly degraded POM in winter. The degradation process metabolizes preferentially lighter isotopes enriching the POM in δ^15^N (Fry, 2006). In consequence, when bottom POM δ^15^N ratios from each region and season were used to calculate the trophic levels of *B. arctica,* these results might artificially decrease their trophic levels, particularly in winter. Alternatively, benthic feeding might also occur, which is known for several mysid species (Mauchline, 1980). Unfortunately, no recent data on the benthos community is available, as the last assessment published is from 2006 (Steinhart, 2023), and since then the hypoxic conditions extended spatially into the GSL and deteriorated severely (Jutras *et al.*, 2023), so that it is unknown to what extend benthic food would be available to *B. arctica*.

Compared to krill species in the EGSL, *B. arctica* was positioned higher than all three krill species in autumn. *M. norvegica* and *T. inermis* were considered as true carnivorous feeder, based on fatty acid and stable isotope ratios (Cabrol *et al.*, 2019a, 2019b), so that carnivorous feeding is also suggested for *B. arctica* in autumn. In contrast, the trophic level of *B. arctica* in winter was comparable to that of *T. raschii,* however, lipid composition was very different, indicating that both species in spite of similar low trophic levels, have a different feeding behavior and physiology.

## CONCLUSION

For the first time, we determined physiological condition, lipid composition and trophic levels of the deep dwelling mysid *B. arctica* in autumn and in winter under sea ice conditions in the Estuary and Gulf of St. Lawrence. *B. arctica* showed high total lipid contents suggesting that *B. arctica* was able to acquire sufficient food to store energy reserves mostly in the form of wax ester particularly in winter. Potential prey organisms such as lipid-rich copepods, in particular, *Calanus*, stay in the deep-water layers of the EGSL during the winter diapause, increasing the encounter probability with *B. arctica*. In view of the biological carbon pump, *B. arctica* potentially acts as an important organism sequestering carbon produced in the upper water layers to the deep layer. In the food web, *B. arctica* is very rich in energetic lipids compared to the three dominant krill species in the EGSL. Therefore, *B. arctica* is probably an interesting prey from an energetic point of view for pelagic and bentho-pelagic consumers that have the enzymatic machinery necessary to digest or assimilate wax esters. However, to better understand the trophic relationships among *B. arctica* and their prey, further quantitative studies (e.g. fatty acid trophic markers and isotopic mixing models) are needed to quantify and better qualify the diet of *B. arctica* and its role in the EGSL ecosystem. Finally, although the aerobic capacities of *B. arctica* are unknown, it appears very interesting that *B. arctica* is still present in similar abundances than 15 years ago, despite the increase of hypoxia in the deep water layer of the Laurentian Channel. Hypoxia might present a restraining environmental variable to this species. However, there is some indication from a congener species *Boreomysis oparva* in the Pacific Ocean that tolerates well hypoxic conditions, even providing a refuge from predators (Saltzman, 1996). In the wake of the environmental changes intensifying in the EGSL, it would be interesting to increase the understanding of the ecology and the fate of this abundant and energy-rich species.

## Supplementary Material

Fig_S1_lipid_classes_nmds_fbae022

## Data Availability

All used datasets for this study including stable isotopes and lipid composition can be found in the SLGO-St. Lawrence Global Observatory or can be provided upon request to the corresponding author.
