# Supplementary figures and images for: Living at depth: ecophysiological condition of Boreomysis arctica in autumn and winter in the St. Lawrence estuary and gulf
Source: J Plankton Res. 2024 May 16;46(3):348–56. doi: 10.1093/plankt/fbae022 (PMC11142453; doi:10.1093/plankt/fbae022)

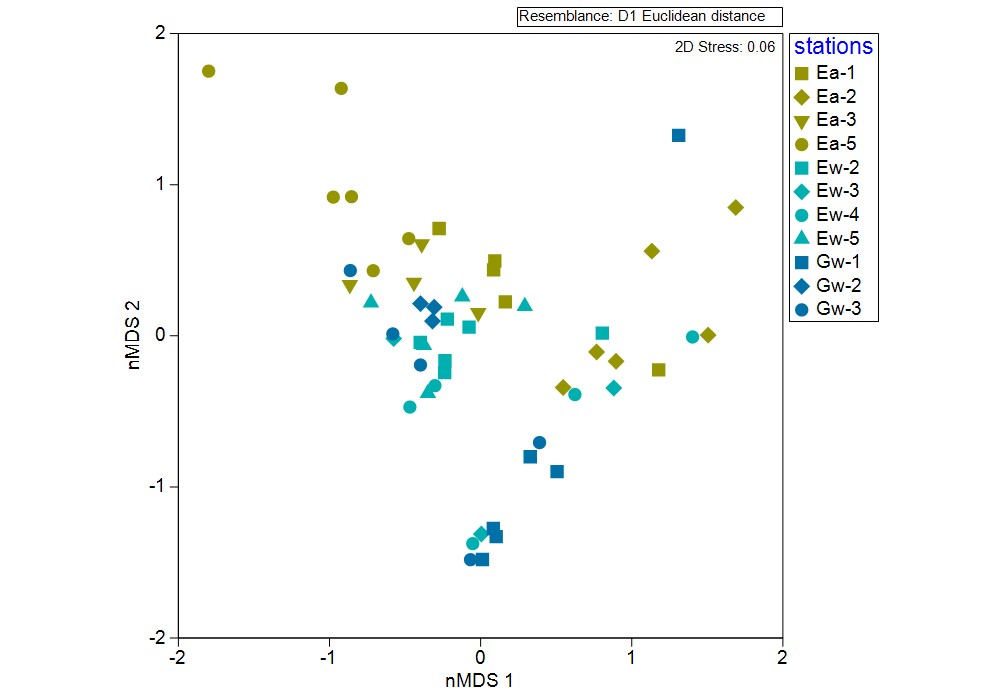

Supplement: Fig_S1_lipid_classes_nmds_fbae022 [file fig_s1_lipid_classes_nmds_fbae022.jpeg]
